# New, fast and cheap prediction tests for *BRCA1* gene mutations identification in clinical samples

**DOI:** 10.1038/s41598-023-34588-9

**Published:** 2023-05-05

**Authors:** Aleksandra Gajda-Walczak, Agnieszka Potęga, Agata Kowalczyk, Slawomir Sek, Sebastian Zięba, Artur Kowalik, Andrzej Kudelski, Anna M. Nowicka

**Affiliations:** 1 Faculty of Chemistry, University of Warsaw, Pasteura 1 Str., 02-093 Warsaw, Poland; 2 Department of Pharmaceutical Technology and Biochemistry, Faculty of Chemistry, Gdańsk University of Technology, Narutowicza 11/12 Str., 80-233 Gdańsk, Poland; 3 Faculty of Chemistry, Biological and Chemical Research Centre, University of Warsaw, Żwirki i Wigury 101 Str., 02-089 Warsaw, Poland; 4 Molecular Diagnostics, Holy Cross Cancer Center, Stefana Artwińskiego 3 Str., 25-734 Kielce, Poland; 5 Division of Medical Biology, Institute of Biology, Jan Kochanowski University, Uniwersytecka 7 Str., 25-406 Kielce, Poland

**Keywords:** Biomarkers, Diagnostic markers, Predictive markers

## Abstract

Despite significant progress in cancer therapy, cancer is still the second cause of mortality in the world. The necessity to make quick therapeutic decisions forces the development of procedures allowing to obtain a reliable result in a quick and unambiguous manner. Currently, detecting predictive mutations, including *BRCA1*, is the basis for effectively treating advanced breast cancer. Here, we present new insight on gene mutation detection. We propose a cheap *BRCA1* mutation detection tests based on the surface plasmon resonance (SPR) or quartz crystal microbalance with energy dissipation (QCM-D) response changes recorded during a hybridization process of an oligonucleotide molecular probe with DNA fragments, with and without the *BRCA1* mutation. The changes in the morphology of the formed DNA layer caused by the presence of the mutation were confirmed by atomic force microscopy. The unique property of the developed SPR and QCM tests is really short time of analysis: *ca.* 6 min for SPR and *ca.* 25 min for QCM. The proposed tests have been verified on 22 different DNA extracted from blood leukocytes collected from cancer patients: 17 samples from patients with various *BRCA1* gene mutation variants including deletion, insertion and missense single-nucleotide and 5 samples from patients without any *BRCA1* mutation. Our test is a response to the need of medical diagnostics for a quick, unambiguous test to identify mutations of the *BRCA1* gene, including missense single-nucleotide (SNPs).

## Introduction

Over the last decade, huge progress has been made in molecular diagnostics, resulting from the development of modern nanotechnology and the introduction of new techniques for DNA analysis, such as next-generation sequencing—which was initially only a research tool, but is now increasingly used in diagnostics, especially in diseases with high genetic heterogeneity^1–6^. For solid tumors (e.g. lung cancer, colorectal cancer, breast cancer and melanoma), it is often difficult to obtain diagnostic material, so other sources of materials are sought that can be easily collected and that provide diagnostic data in order to regularly monitor the effectiveness of treatment. One material having such features is the peripheral blood, which reaches all cells of the body^7^. Diagnostics based on peripheral blood is called liquid biopsy^8,9^. Liquid biopsy makes it possible to study genetic material isolated from the blood, such as circulating tumor cells (CTCs). This method also makes it possible to analyze the circulating free DNA (cfDNA) present outside the cells. In healthy people, the amount of cfDNA is very small, but it increases significantly in cancer patients. A tumor releases tumor cells (CTC) into the bloodstream. These undergo apoptosis or necrosis, and release free circulating tumor DNA (ctDNA) into the bloodstream^10,11^. CtDNA analysis is believed to reveal the genetic abnormality present in each tumor in the patient's body. CtDNA is present in 50–75% of patients with non-advanced or advanced cancer (e.g. pancreatic, breast, intestinal, kidney and brain cancers)^12^. CtDNA testing can therefore provide reliable information about the cancer status, regardless of the nature of the cancer or the type of treatment. The great advantage of ctDNA analysis over radiological methods is the possibility it offers of detecting disease progression much earlier—even up to 10 months earlier^13^. The challenge of the method is the small amount of ctDNA released into the bloodstream and the lack of ultra-sensitive diagnostic methods capable of reliably detecting 0.1–0.01% of the mutant allele, especially bearing in mind that, in the early stages of cancer, there are only trace amounts of biomarkers in the blood of patients with solid tumors.

One of the most common malignancies in women around the world, and one of the major causes of death among women, is breast cancer^14^. Epidemiological studies indicate that about 5–10% of all breast cancer cases in women are linked to hereditary susceptibility due to mutations in autosomal dominant genes with high penetrance^15^. The genetic variations found in this kind of cancer are usually loss-of-function mutations in tumor suppressor genes which result in uncontrollable cell growth, inability to repair DNA after damage, and a lack of cell cycle check-points^16,17^. Two key factors associated with a high risk of breast cancer are mutations in the *BRCA1* and *BRCA2* (Breast Cancer; OMIM 113705 and OMIM 600185, respectively) genes^18,19^, with the majority of hereditary cases attributed to the *BRCA1* gene^20,21^. Moreover, women with mutations in the *BRCA* gene are at increased risk of developing ovarian cancer, which is one of the most lethal cancers^22^. *BRCA1* protein plays a pivotal role in maintaining genomic stability through transcriptional regulation and DNA repair^23^. According to the Breast Information Core (BIC), most of the breast cancer-causing mutations in the *BRCA1* gene lead to the production of a truncated protein that fails to perform its physiological functions^24^. These genetic alternations mainly include small frame shifts, nonsense mutations, splice-site mutations, and deletions. Small insertions, missense mutations, and a premature transcription termination have also been identified^17,25^. Due to the high mortality and morbidity of breast and ovarian cancers worldwide, the rapid and efficient detection of *BRCA1* gene mutant variants is of great significance for predicting the risk of cancer. It can also help clinicians and patients make informed decisions about preventive treatments. However, the large size of the *BRCA1* gene, and diversity of mutations, make such analysis complex and challenging.

The gold standard in *BRCA1* gene mutation diagnostic is direct sequence analysis^26^. However, this approach is both time-consuming and costly^27^. Other faster, cheaper alternative strategies rely on scanning techniques to identify *BRCA1* gene fragments containing genetic sequence changes, but do not provide information on the type of mutation. The most commonly used scanning techniques are: single-strand conformation polymorphism (SSCP), conformation-sensitive gel electrophoresis (CSGE), fluorescence-based conformation-sensitive gel electrophoresis (F-CSGE), the protein truncation test (PTT), high-resolution melting analysis (HRM), and denaturing high performance liquid chromatography (DHPLC)^26,28,29^. These techniques are sensitive enough to detect a low level of the mutation, but none of them can be used to detect large rearrangements such as deletions of whole exons^30^.

Taking account of the complications related to the amount of genetic material obtained and the expected sensitivity, new methods with high sensitivity and good specificity are still needed. In this work, we developed a novel, fast and simple predictive test using surface plasmon resonance (SPR) or quartz crystal microbalance with energy dissipation (QCM-D) detection to identify *BRCA1* mutations based on the DNA hybridization protocol. The studies were performed both on synthetic oligonucleotides and real samples collected from patients. Applying two detection techniques based on affinity binding of the analyte (target DNA) to the receptor (probe DNA) immobilized on the gold surface significantly authenticates the correctness of the obtained results. Importantly, these techniques do not require the presence of any reporter, which significantly simplifies the detection process. Our proposition may be an excellent alternative to the molecular diagnostics methods in current use.

## Materials and methods

### Materials

Ethylenediaminetetraacetic acid disodium salt (EDTA; Sigma Aldrich), mercapto-1-hexanol (MCH; Sigma Aldrich), phosphate-buffered saline (PBS, 10×; Sigma Aldrich), sodium acetate (NaAc; POCH), tris(2–carboxyethyl)phosphine hydrochloride (TCEP; Sigma Aldrich), magnesium acetate (Mg(Ac)_2_; POCH), 2–amino–2–(hydroxymethyl)propane–1,3–diol (Tris; Sigma Aldrich), absolute ethanol (99.8%; POCH) were of the highest purity available. All of the oligonucleotides were purchased from MWG–Operon (Eurofins). The oligonucleotide sequences used are presented in Table 1. The thiolated probe DNA fragments were synthesized as a disulfide. To reduce the disulphide bond to the sulfhydryl group, the thiolated DNA sequences were dissolved in 200 μL of 10 mM TCEP in a TE buffer (10 mM Tris, 1 mM EDTA, pH 8.0) and shaken at room temperature for 60 min using a ThermoMixer (Eppendorf). The probe DNA sequences were precipitated by adding 150 μL of a mixture containing 3 M NaAc and 1 mM of Mg(Ac)_2_. Next, the tubes were filled with absolute ethanol, gently shaken, and incubated at  − 20 °C for 20 min. Next, the mixtures were centrifuged at 13,000 rpm for 5 min, and finally the pellets were left to dry at room temperature. The dried pellets were diluted in distilled water to a concentration 100 μM. Before each experiment, all the DNA sequences, diluted to appropriate concentration, were heated to melting temperature (given by the manufacturer) for 10 min. and then immediately cooled down in an ice bath.

**Table 1:** Used synthetic DNA sequences.

*BRCA1* gene mutation: 5370C > T (gene fragment at position c.5251 C > T transition)	
Without mutation	probe DNA 1 (5′ → 3′): thiol–C_6_–AGGTCCAAAG**C**GAGCAAGAGAAGGAGTGGGTCCCATCAGTTTGAA
	target DNA 1 (3′ → 5′): TCCAGGTTTC**G**CTCGTTCTCTTCCTCACCCAGGGTAGTCAAACTT
With mutation	probe DNA 2 (5′ → 3′): thiol–C_6_–AGGTCCAAAG**T**GAGCAAGAGAAGGAGTGGGTCCCATCAGTTTGAA
	target DNA 2 (3′ → 5′): TCCAGGTTTC**A**CTCGTTCTCTTCCTCACCCAGGGTAGTCAAACTT
*BRCA1* gene mutation: 5382insC (gene fragment at position c.5266_67)	
Without mutation	probe DNA 3 (5′ → 3′): thiol–C6–AAGAGAATCCC☐AGGACAGAAAGGGAGTGGGTCCCATCAGTTTGAA
	target DNA 3 (3′ → 5′): TTCTCTTAGGG☐TCCTGTCTTTCCCTCACCCAGGGTAGTCAAACTT
With mutation	probe DNA 4 (5′ → 3′): thiol–C_6_–AAGAGAATCCC**C**AGGACAGAAAGGGAGTGGGTCCCATCAGTTTGAA
	target DNA 4 (3′ → 5′): TTCTCTTAGGG**G**TCCTGTCTTTCCCTCACCCAGGGTAGTCAAACTT
*BRCA1* gene mutation: c.4035delA (gene fragment at position c.4035)	
Without mutation	probe DNA 5 (5′ → 3′): thiol–C_6_–TCAGATGATGA**A**GAAAGAGGAACGGAGTGGGTCCCATCAGTTTGAA
	target DNA 5 (3′ → 5′): AGTCTACTACT**T**CTTTCTCCTTGCCTCACCCAGGGTAGTCAAACTT
With mutation	probe DNA 6 (5′ → 3′): thiol–C6–TCAGATGATGA☐GAAAGAGGAACGGAGTGGGTCCCATCAGTTTGAA
	target DNA 6 (3′ → 5′): AGTCTACTACT☐CTTTCTCCTTGCCTCACCCAGGGTAGTCAAACTT
*BRCA1* gene mutation: 185delAG (gene fragment at position c.68_69)	
Without mutation	probe DNA 7 (5′ → 3′): thiol–C_6_–CAGAAAATCTTAG**AG**TGTCCCATGGAGTGGGTCCCATCAGTTTGAA
	target DNA 7 (3′ → 5′): GTCTTTTAGAATC**TC**ACAGGGTACCTCACCCAGGGTAGTCAAACTT
With mutation	probe DNA 8 (5′ → 3′): thiol–C_6_–CAGAAAATCTTAG☐☐TGTCCCATGGAGTGGGTCCCATCAGTTTGAA
	target DNA 8 (3′ → 5′): GTCTTTTAGAATC☐☐ACAGGGTACCTCACCCAGGGTAGTCAAACTT
*BRCA1* gene mutation: 3819del5GTAAA (gene fragment at position c.3700_3705)	
Without mutation	probe DNA 9 (5′ → 3′): thiol–C_6_–ATTTGGTAAA**GTAAA**CAATATACCTGGAGTGGGTCCCATCAGTTTGAA
	target DNA 9 (3′ → 5′): TAAACCATTT**CATTT**GTTATATGGACCTCACCCAGGGTAGTCAAACTT
With mutation	probe DNA 10 (5′ → 3′): thiol–C6–ATTTGGTAAA☐☐☐☐☐CAATATACCTGGAGTGGGTCCCATCAGTTTGAA
	target DNA 10 (3′ → 5′): TAAACCATTT☐☐☐☐☐GTTATATGGACCTCACCCAGGGTAGTCAAACTT

### Clinical samples

The DNA samples were isolated from blood taken from 22 patients under the care of the Holy Cross Cancer Centre Genetic Outpatient Clinic, for whom routine testing of mutations occurring in the *BRCA1* gene was performed using high-resolution melting (HRM) and Sanger sequencing. The DNA samples were used after obtaining the informed consent of the patients to the use of their biological material for scientific purposes. DNA was isolated from 300 μL whole blood with Maxwell^®^ RSC Whole Blood DNA Kit (Promega, Madison, WI, USA) using Maxwell^®^ RSC Instrument (Promega, Madison, WI, USA) according to manufacturer protocol.

Two microliters of isolated DNA were used for concentration measurement with Qubit dsDNA BR (broad range) Assay Kit and Qubit 3 (Thermo Fisher Scientific, Waltham, MA, USA) according to manufacturer instruction. The mean DNA concentration was 25 ng μL^−1^. Of the 22 DNA samples selected for testing, 18 had a detectable mutation in the *BRCA1* gene:

- 10 × mutation 5382insC [c.5266dup; p.(Gln1756Profs*74)],
- 1 × mutation 5370C > T [c.5251C > T; p.(Arg1751*)],
- 1 × c.68_69delAG mutation [c.68_69del; p.(Glu23Valfs*17)],
- 2 × c.4035delA mutation [c.4035del; p.(Glu1346Lysfs*20)],
- 3 × mutation of c. 3819del5) [c.3700_3704del; p.(Val1234Glnfs*8)],
- and 5 samples without any of the above mutations detected.

The above variants, presented in Table 2, are well known founder mutations in *BRCA1* for the Polish population. Before the experiments, the regions covering the above-mentioned mutations were amplified by PCR using the same primers as for diagnostic HRM. The PCR was performed using a Veriti Thermal Cycler (Applied Biosystems, Foster City, California, USA). The reactions were carried out in a final volume of 20 μL containing 7 μL Mix Qiagen Type-it HRM PCR (Qiagen, Hilden, Germany), 5 μL H_2_O, 1 μL of each primer, and 1 μL genomic DNA. The PCR conditions were as follows: 95 °C for 5 min., 10 cycles of 95 °C for 10 s, 62.5 °C for 30 s (+ touchdown 10 × 1°), 72 °C for 20 s, and 30 cycles of 95 °C for 10 s, 57 °C for 30 s, 72 °C for 20 s. All the study procedures were approved by the local Bioethics Commission affiliated with the Holy Cross Region Chamber of Physicians and performed according to the Declaration of Helsinki. All patients provided signed, informed consent before enrolling in the study. Only the samples of blood remaining after the standard clinical procedures performed in Holy Cross Cancer Center were used.

**Table 2:** List of primers used for PCR amplification. The reference sequence NM_007294.3 was used for the *BRCA1*.

*BRCA1*	Primer name	Primer sequence (5′ → 3′)	Amplicon size
Exon 2	*BRCA1*_ek2_F_HRM	GAAGTTGTCATTTTATAAACCTTT	258 bp
	*BRCA1*_ek2_R_HRM	TGTCTTTTCTTCCCTAGTATGT	
Exon 4	*BRCA1*_ek5_F_HRM	CTCTTAAGGGCAGTTGTGAG	235 bp
	*BRCA1*_ek5_R_HRM	TTCCTACTGTGGTTGCTTCC	
Exon 10	*BRCA1*_ek11_F_HRM	CAGGGAGTTGGTCTGAGTGAC	181 bp
	*BRCA1*_ek11_R_HRM	GCTCCCCAAAAGCATAAAC	
Exon 19	*BRCA1*_ek20_F_HRM	ATATGACGTGTCTGCTCCAC	401 bp
	*BRCA1*_ek20_R_HRM	GGGAATCCAAATTACACAGC	

### Surface plasmon resonance (SPR)

The SPR measurements were performed using a Biacore X100 system (GE Healthcare) from Cytiva (Uppsala, Sweden), on an Au chip (Cytiva). Before the experiments, the gold chip was cleaned in accordance with the TL1 procedure. A mixture of ultrapure water, 25% ammonia and 30% hydrogen peroxide at a volume ratio 5:1:1 was heated to a temperature of 75 °C, after which the gold chip was immersed in this mixture for 5 min. Next, the surface of the Au-chip was rinsed with ultrapure water and then with 99.8% ethanol and dried with argon. The freshly cleaned SPR gold chip was placed in measurement chamber. After a stable baseline was achieved in a PBS buffer, the probe DNA solution (100 nM in 0.01 M PBS with the addition of 1 mM EDTA and 1 M NaCl) was injected onto the chip surface (contact time: 600 s; flow rate 5 μL min^−1^). The probe immobilization was followed by the addition of 1 μM solution of MCH in 0.01 M PBS with the addition of 1 mM EDTA and 1 M NaCl (contact time: 200 s; flow rate 5 μL min^−1^). Finally, a hybridization process was performed with the appropriate target DNA sequence in 0.01 M PBS with the addition of 1 mM EDTA and 1 M NaCl (contact time: 300 s; dissociation time: 600 s; flow rate 5 μL min^−1^). After the hybridization process with the target DNA (synthetic or clinical sequence), the surface of the Au chip was regenerated in two steps: (i) 60-s of injection of 1 M NaCl (flow rate 10 μL min^−1^), (ii) 30-s of injection of 50 mM NaOH (flow rate 10 μL min^−1^).

### Quartz crystal microbalance with dissipation (QCM-D)

*BRCA1* mutations were also detected using a quartz crystal microbalance with dissipation (QCM-D; E4 model from Q-Sense, Biolin Scientific) equipped with 4.95 MHz AT-cut gold-coated quartz crystals. The measurements were carried out in a flow system at a flow rate of 60 μL min^−1^ at room temperature (21 °C). Before the experiments, the gold-coated quartz crystals were cleaned in accordance with the same procedure used with the SPR Au chips. Due to the strong adsorption of thiol compounds, the preparation of the receptor layer (Au/probe DNA/MCH) was carried out outside the QCM-D system.

### Atomic force microscopy (AFM)

The imaging was performed using a Dimension Icon (Bruker, Santa Barbara, CA) instrument. The images were collected in an aqueous solution of 0.01 M PBS with the addition of 1 mM EDTA and 1 M NaCl. ScanAsyst Fluid probes (Bruker, Santa Barbara, CA) with a nominal spring constant of 0.7 N m^−1^ were used to collect images of the morphology of the samples. The probes were calibrated before each experiment using the thermal tune method. Au(111) single crystals were used as substrates. Single-crystal Au(111) substrate (diameter of 10 mm, orientation accuracy of < 0.1°) was purchased from MaTecK GmbH (Jülich, Germany). Before each measurement, the Au(111) discs were cleaned in a piranha solution (a mixture of H_2_SO_4_ and H_2_O_2_, volume ratio 3:1), and were then thoroughly washed with ultrapure water and flame-annealed. Next, the substrates were modified in accordance with the same protocol as described before, with the exception that static conditions were used (instead of flow) and the washing step with the buffer was applied after the adsorption of thiolated DNA and MCH.

## Results and discussions

The need to make quick therapeutic decisions requires procedures that allow reliable results to be obtained quickly and unambiguously. Such an ideal genetic test for detecting predictive mutations, i.e. those that already have a defined therapeutic path, should be characterized above all by the reliability of their results, simplicity, a short implementation time, low cost, and the use of only a small amount of diagnostic material. Most of the tests available on the market are based on the polymerase chain reaction, but unfortunately, such tests are expensive, time-consuming and must be carried out by qualified personnel. There is still a need, then, for fast and cheap mutation identification tools. In this work, we have proved that SPR and QCM-D perfectly fill the gap in what is expected from diagnostic tests for gene mutations.

### SPR analysis

**Figure 1:**
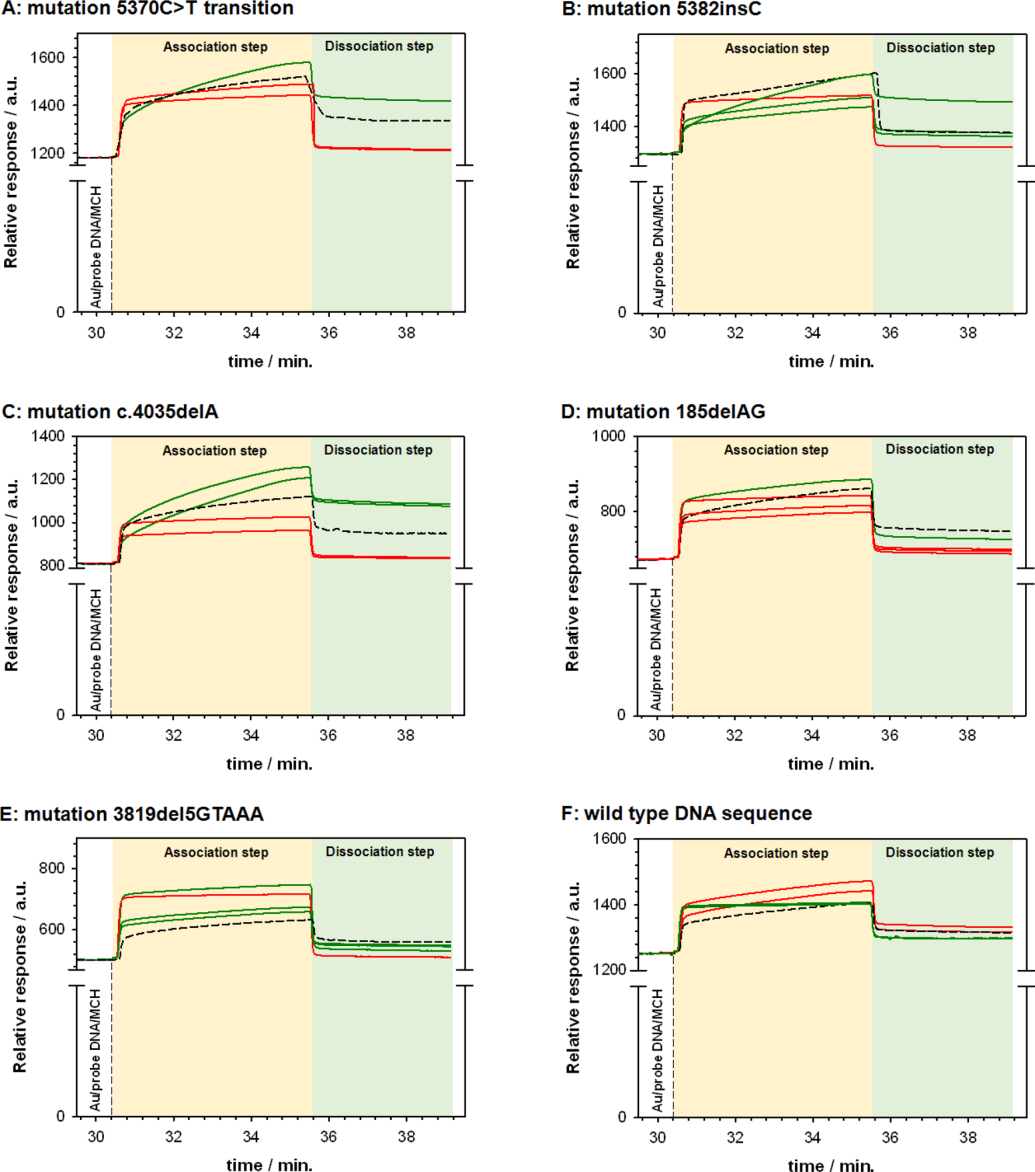
SPR sensorgrams recorded during the hybridization process with target DNA sequences: synthetic (dashed lines), obtained from patients with (green lines) and without (red lines) appropriate mutations (**A**–**E**) and for wild type DNA sequence (**F**). Experimental conditions: 0.01 M PBS with the addition of 1 mM EDTA and 1 M NaCl; *C*_probe DNA_ = 100 nM (contact time: 600 s; flow rate 5 μL min^−1^); *C*_MCH_ = 1.0 μM (contact time: 200 s; flow rate 5 μL min^−1^); *C*_synthetic target DNA_ = 100 nM (0.15 ng μL^−1^) or 200-fold diluted clinical sample (*C*_DNA in clinical samples_ = 0.13 ng μL^−1^), contact time: 300 s, dissociation time: 600 s, flow rate 5 μL min^−1^.

Surface plasmon resonance is an optical effect that can be used to study the bonding of molecules in real time, without labeling. Using this technique, we can obtain information about the amount of the substance sought in the analyzed sample, and about the specificity and selectivity of the complex formed. In this work, SPR measurements were performed on samples with and without the *BRCA1* mutation. The resulting sensorgrams are shown in Fig. 1.

The procedure for identifying mutations consisted of three stages. In the first stage, as a result of the self-assembly process, single probe DNA fragments characteristic of each type of *BRCA1* mutation were attached to the surface of the gold chip. The next step was to seal the receptor layer: the sealing thiol should effectively protect the probe DNA fragments against becoming adsorbed on the gold substrate, but should not in any way impede the hybridization process. For this purpose, mercaptothiol (MCH) was used; its length was consistent with the length of the carbon linker present in the probe DNA. The last step concerned the detection of the DNA sequence complementary to the probe DNA. The sensorgrams recorded at each of these stages contain two components that describe the association and dissociation processes. In the association phase, the DNA fragments present in the analyzed sample undergo a hybridization process with the probe DNA immobilized on the gold chip surface. The effectiveness of this process depends on the strength of the interactions between the target DNA and the probe DNA (their degree of complementarity). As a result of the formation of a DNA double helix, the signal increases until equilibrium is reached (if this is achieved), when the maximum number of analyte molecules has bound to the receptor layer. Replacing the analyte solution with the buffer results in a reverse process (the dissociation phase), in which the complex formed degrades. The intensity of the SPR signal then begins to decrease. The relationships describing the phases of association and dissociation should be exponential. Analyzing the obtained sensorgrams, we see that such an exponential character was obtained only when the analyte (target DNA) was fully complementary to the DNA probe (the curves marked in Fig. 1A–E with black dashed lines (synthetic sequences) and green lines (real samples)). Even a partial lack of complementarity with the target DNA (the red curves in Fig. 1A–E) resulted in a drastic change in the nature of the curve—the increase of the SPR signal was much smaller and slower. It should be emphasized that, even in the case of a point mutation (5370C > T, see in Fig. 1A), the SPR relationship obtained was significantly different from that recorded for the samples containing a wild type (WT) DNA sequence. This observation confirms the specificity of the developed test operation. Control experiments with the probe DNA corresponding sequence without mutation were also performed. The character of the relationships obtained, SPR response = f(*t*) was identical; a significant exponential increase in the SPR signal was only observed when there was a fully complementary DNA sequence (Fig. 1F). It should be noted that in the kinetic studies the synthetic target DNA concentration as well as the DNA concentration in clinical samples was very similar and equaled 0.15 and 0.13 ng μL^−1^, respectively. To obtain detailed information from the SPR measurements, a simple model of 1:1 binding $\left( {{\text{probe DNA + target DNA}}\underset{{\text{kd, Kd}}}{\overset{{\text{ka, Ka}}}{\leftrightarrows}}{\text{ double stranded DNA}}} \right)$ was selected for fitting the experimental results to determine the values of the association (*k*_a_) and dissociation (*k*_d_) rate constants. The values of dissociation (*K*_d_) and association (*K*_a_) equilibrium constants were determined from following equations:

1 $${{K}}_{\text{d}}\text{=}\frac{{k}_{\text{d}}}{{k}_{\text{a}}}$$

2 $$ {{K}}_{\text{a}}\text{=}\frac{1}{{{K}}_{\text{d}}}$$

Table 3 presents the kinetic parameters (*k*_a_: association rate constant; *k*_d_: dissociation rate constant; *K*_a_: association equilibrium constant;* K*_d_: dissociation equilibrium constant) determined from SPR experiments. Evidently, the association rate constants for DNA duplex formation step were at least 10^3^–10^4^ times higher, when DNA fragments present in the analyzed solution were fully complementary to the oligonucleotide sequence immobilized on the gold surface than for non-fully-complementary DNA fragments. Whereas, the values of the association equilibrium constants clearly show that a strong affinity between the DNA fragments (probe DNA : target DNA) was observed only when the DNA strands were completely complementary to each other. Where there was a partial lack of complementarity, the values of the dissociation constants were at least 3–4 orders of magnitude lower. Taking into account the above information, it can be concluded that the threshold value of the association equilibrium constant (*K*_a_) allowing for the classification of a sample to a group of samples containing a given mutation or to a group of mutation-free samples should be a maximum of an order of magnitude lower than the value determined for fully complementary target synthetic DNA.

**Table 3:** Kinetic parameters (*k*_a_: association rate constant; *k*_d_: dissociation rate constant; *K*_a_: association equilibrium constant;* K*_d_: dissociation equilibrium constant) obtained from SPR data for the hybridization process with target DNA sequences: synthetic, and obtained from patients with and without appropriate mutations.

Mutation	Type of sample	Sequence of *BRCA1* gene without mutation				Sequence of *BRCA1* gene with mutation			
		*k*_a_ [M^−1^ s^−1^]	*k*_d_ [s^−1^]	*K*_d_ [M]	*K*_a_ [M^−1^]	*k*_a_ [M^−1^ s^−1^]	*k*_d_ [s^−1^]	*K*_d_ [M]	*K*_a_ [M^−1^]
5370 C > T transition	Target	2.61 × 10^7^	8.09 × 10^−3^	3.11 × 10^−10^	3.22 × 10^9^	1.45 × 10^6^	2.87 × 10^−4^	1.98 × 10^−10^	5.05 × 10^9^
	Patient 1 (with mut.)	5.40 × 10^3^	1.36 × 10^−3^	2.52 × 10^−7^	3.97 × 10^6^	6.62 × 10^5^	3.04 × 10^−4^	4.59 × 10^−10^	2.18 × 10^9^
	Patient 2 (with mut.)	1.31 × 10^4^	5.59 × 10^−3^	4.27 × 10^−7^	2.34 × 10^6^	3.14 × 10^5^	1.89 × 10^−4^	6.02 × 10^−10^	1.66 × 10^9^
	Patient 3 (without mut.)	1.44 × 10^7^	7.25 × 10^−3^	5.04 × 10^−10^	1.98 × 10^9^	7.53 × 10^3^	6.41 × 10^−3^	8.51 × 10^−7^	1.18 × 10^6^
	Patient 4 (without mut.)	3.11 × 10^7^	6.13 × 10^−3^	1.97 × 10^−10^	5.08 × 10^9^	5.93 × 10^5^	5.99 × 10^−3^	1.01 × 10^−8^	9.90 × 10^7^
5382insC	Target	1.71 × 10^6^	1.21 × 10^−3^	7.06 × 10^−10^	1.42 × 10^9^	9.72 × 10^7^	4.26 × 10^−3^	4.38 × 10^−11^	2.28 × 10^10^
	Patient 5 (with mut.)	6.80 × 10^2^	7.62 × 10^−3^	1.12 × 10^−5^	8.93 × 10^4^	3.84 × 10^8^	5.31 × 10^−3^	1.38 × 10^−11^	7.25 × 10^10^
	Patient 6 (with mut.)	1.19 × 10^2^	3.25 × 10^−3^	2.72 × 10^−5^	3.68 × 10^4^	2.33 × 10^6^	1.04 × 10^−4^	4.46 × 10^−11^	2.24 × 10^10^
	Patient 7 (with mut.)	2.33 × 10^2^	9.82 × 10^−3^	4.21 × 10^−5^	2.38 × 10^4^	3.64 × 10^8^	7.82 × 10^−3^	2.15 × 10^−11^	4.65 × 10^10^
	Patient 8 (without mut.)	5.04 × 10^6^	3.21 × 10^−3^	6.37 × 10^−10^	1.57 × 10^9^	1.51 × 10^2^	3.26 × 10^−4^	2.15 × 10^−6^	4.65 × 10^5^
c.4035delA	Target	1.06 × 10^6^	2.82 × 10^−4^	2.66 × 10^−10^	3.76 × 10^9^	8.17 × 10^6^	3.86 × 10^−4^	4.72 × 10^−11^	2.12 × 10^10^
	Patient 9 (with mut.)	3.73 × 10^2^	1.20 × 10^−3^	3.21 × 10^−6^	3.12 × 10^5^	3.03 × 10^6^	1.89 × 10^−4^	6.24 × 10^−11^	1.60 × 10^10^
	Patient 10 (with mut.)	4.91 × 10^2^	2.76 × 10^−3^	5.62 × 10^−6^	1.78 × 10^5^	1.65 × 10^6^	7.21 × 10^−4^	4.37 × 10^−10^	2.29 × 10^9^
	Patient 11 (without mut.)	2.68 × 10^6^	4.35 × 10^−4^	1.62 × 10^−10^	6.17 × 10^9^	4.69 × 10^2^	4.32 × 10^−3^	9.21 × 10^−6^	1.09 × 10^5^
	Patient 12 (without mut.)	2.65 × 10^6^	3.26 × 10^−4^	1.23 × 10^−10^	8.13 × 10^9^	1.27 × 10^3^	7.93 × 10^−3^	6.24 × 10^−6^	1.60 × 10^5^
185delAG	Target	2.04 × 10^5^	1.46 × 10^−5^	7.14 × 10^−11^	1.40 × 10^10^	4.22 × 10^6^	5.23 × 10^−4^	1.24 × 10^−10^	8.06 × 10^9^
	Patient 13 (with mut.)	4.68 × 10^2^	2.33 × 10^−3^	4.98 × 10^−6^	2.01 × 10^5^	2.65 × 10^6^	3.19 × 10^−4^	1.20 × 10^−10^	8.33 × 10^9^
	Patient 14 (without mut.)	2.97 × 10^5^	1.73 × 10^−5^	5.85 × 10^−11^	1.71 × 10^10^	3.43 × 10^3^	5.63 × 10^−2^	1.64 × 10^−5^	6.10 × 10^4^
	Patient 15 (without mut.)	3.02 × 10^5^	3.45 × 10^−5^	1.14 × 10^−10^	8.77 × 10^9^	8.81 × 10^2^	6.27 × 10^−3^	7.12 × 10^−6^	1.40 × 10^5^
	Patient 16 (without mut.)	2.93 × 10^5^	2.97 × 10^−5^	1.01 × 10^−10^	9.90 × 10^9^	4.22 × 10^3^	6.19 × 10^−2^	1.47 × 10^−5^	6.80 × 10^4^
3819del5GTAAA	Target	2.32 × 10^6^	4.58 × 10^−3^	1.97 × 10^−9^	5.08 × 10^8^	1.05 × 10^7^	7.21 × 10^−3^	6.84 × 10^−10^	1.46 × 10^9^
	Patient 17 (with mut.)	5.56 × 10^1^	2.41 × 10^−3^	4.33 × 10^−5^	2.31 × 10^4^	1.73 × 10^7^	8.38 × 10^−3^	4.84 × 10^−10^	2.07 × 10^9^
	Patient 18 (with mut.)	1.51 × 10^3^	4.85 × 10^−3^	3.21 × 10^−6^	3.12 × 10^5^	3.81 × 10^7^	6.72 × 10^−3^	1.76 × 10^−10^	5.68 × 10^9^
	Patient 19 (with mut.)	5.15 × 10^1^	3.72 × 10^−3^	7.21 × 10^−5^	1.39 × 10^4^	2.39 × 10^7^	7.77 × 10^−3^	3.24 × 10^−10^	3.09 × 10^9^
	Patient 20 (without mut.)	2.58 × 10^7^	5.31 × 10^−2^	2.06 × 10^−9^	4.85 × 10^8^	1.14 × 10^1^	4.25 × 10^−4^	3.71 × 10^−5^	2.70 × 10^4^

The DNA sequences concentration: *C*_synthetic target DNA_ = 0.15 ng μL^−1^; *C*_DNA in clinical samples_ = 0.13 ng μL^−1^.

### QCM-D analysis

**Figure 2:**
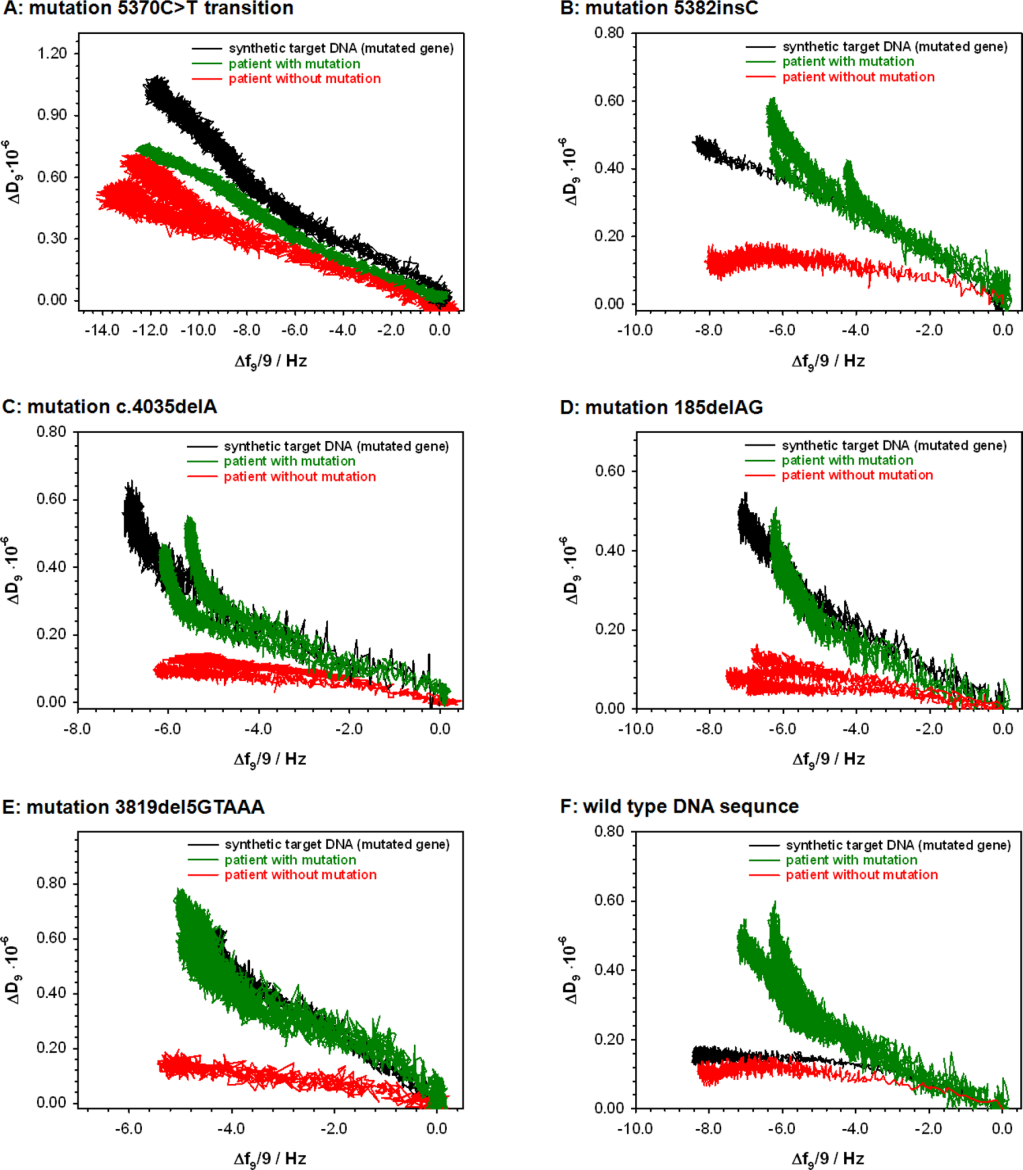
Relationships Δ*D* = f(Δ*f*) recorded during the hybridization process with target DNA sequences: synthetic mutated *BRCA1* gene sequence (black lines), sequences obtained from patients with (green lines) and without (red lines) appropriate mutations (**A**–**E**) and wild type DNA sequence (**F**). Experimental conditions: 0.01 M PBS with the addition of 1 mM EDTA and 1 M NaCl; *C*_probe DNA_ = 100 nM (*V*_droplet_ = 100 μL, *t* = 2 h); *C*_MCH_ = 1.0 μM (*V*_droplet_ = 100 μL, *t* = 1 h); *C*_synthetic target DNA_ = 100 nM (0.15 ng μL^−1^) or 200-fold diluted clinical sample. (*C*_DNA in clinical samples_ = 0.13 ng μL^−1^).

Another technique that allows for real-time control over the interactions between molecules without labeling is quartz crystal microbalance with dissipation. This technique, like SPR, makes it possible to obtain information about the specificity of the complex formed. A typical relationship recorded using this technique describes the changes in the frequency energy dissipation coefficient of the quartz crystal and as a function of time, see Fig. 1S in Supporting Information. The synthetic target DNA concentration as well as the DNA concentration in clinical samples applied during the hybridization process was very similar and equaled 0.15 and 0.13 ng μL^−1^, respectively. Determining the parameters of the analyte-receptor interaction on the basis of the frequency changes of the quartz crystal is possible only when the analyte is precisely defined (its structure, molecular weight, etc. are known). In the case of the clinical samples, the exact length of the DNA fragments present in the solution is unknown; such DNA fragments, in addition to the target sequence, is also enriched in other nucleotides that the synthetic target sequence (standard) does not contain. In such situation, the recognition of the target DNA with QCM-D detector should be based on the Δ*D* = f(Δ*f*) relationship, which is a much better diagnostic parameter. In the case of the presence of a correct (without mutation) DNA sequence in the analyzed solution, the DNA strands formed as a result of the recognition process were only partially hybridized. The as-formed hybrid: probe DNA-target DNA is only fragmentarily a double helix. This situation must be reflected in the morphology of the formed DNA layer—in its organization, packing density and regularity. The degree to which the DNA fragments match each other in accordance with the complementarity principle is very well illustrated by the relationships Δ*D* = f(Δ*f*), presented in Fig. 2. These relationships clearly show the organization of the layer, where the change in slope indicates changes in the regularity and packing density of the layer formed on the quartz crystal surface. As can be seen in Fig. 2A–E, only when the analyzed sample contained fragments that were fully complementary to the probe DNA fragments modifying the quartz crystal surface was the nature of these changes identical to those obtained for the synthetic complementary DNA strand. In the presence in the analyzed solution of not fully complementary DNA fragments, the slope of the relationship was significantly lower than the reference curve (obtained for the fully complementary synthetic target DNA). Unfortunately, gravimetric detection does not make it possible to detect a point mutation (5370C > T, see Fig. 2A). The relationships Δ*D* = f(Δ*f*) recorded for the mutated and non-mutated samples were very similar to each other. Similar relationships were observed when a wild type DNA sequence of the *BRCA1* gene was used as the probe DNA (see Fig. 2F). In this situation, agreement between the reference curve (the black curve in Fig. 2F) and the obtained result was found only when the DNA sequence from the patient without the mutated gene (the red curve in Fig. 2F) was present in the solution.

### AFM analysis

**Figure 3:**
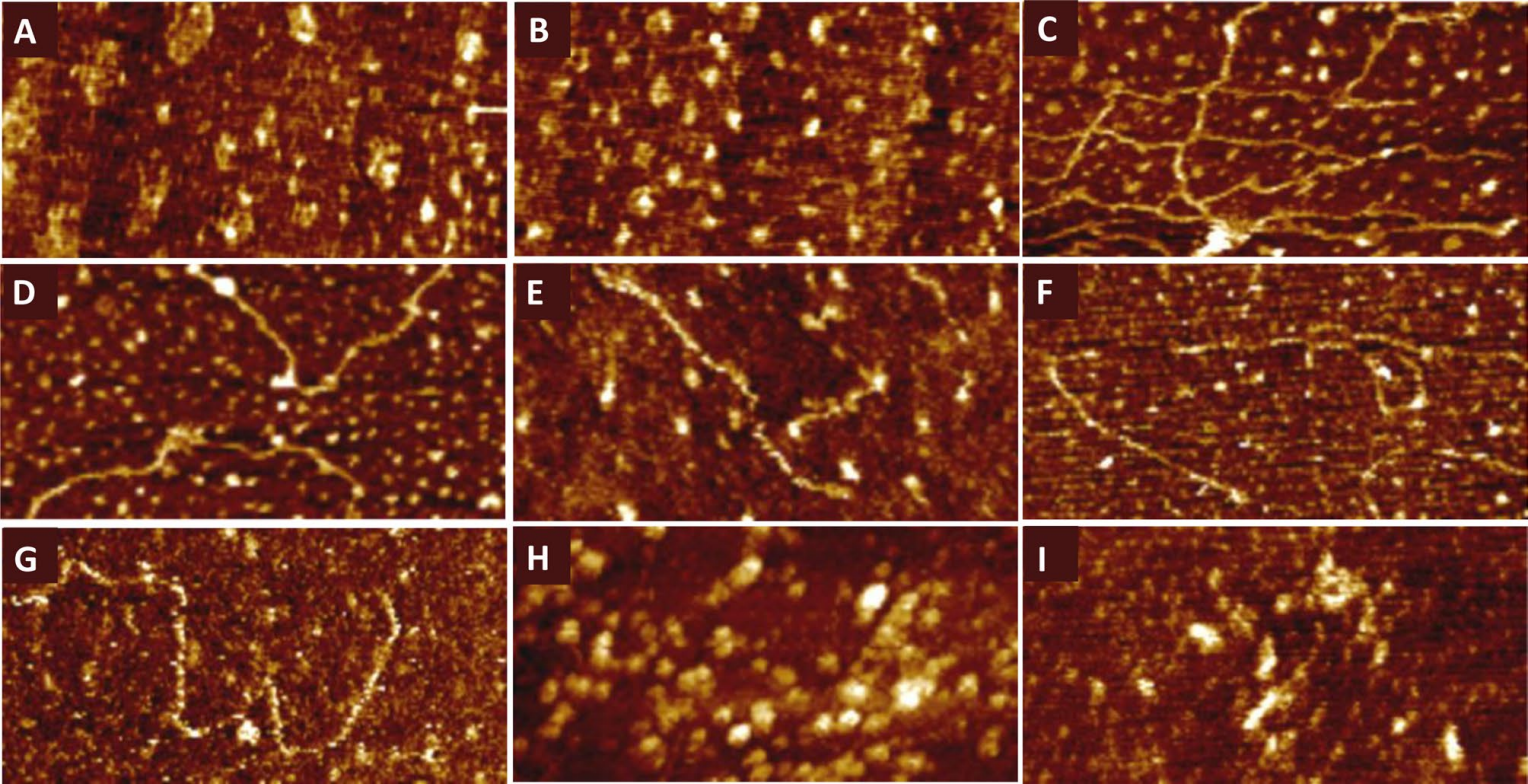
AFM images recorded for an Au(111) substrate subsequently modified with the probe DNA (**A**), MCH (**B**) and further exposed for 2 h to the target DNA (synthetic fully complementary to the probe DNA) (**C**); and clinical samples containing: variant 5382insC (**D**); variant 3819del5GTAAA (**E**); variant 5370C > T (**F**); variant c.4035delA (**G**); wild type (**H**); wild type (**I**). The size of the images is 400 × 200  nm^2^.

The morphology of the films deposited on the gold surface was analyzed based on AFM images obtained in an aqueous solution of 0.01 M PBS with the addition of 1 mM EDTA and 1 M NaCl. Sample images are presented in Fig. 3. After initial modification with the thiolated probe DNA (see Fig. 3A), irregularly shaped islands of the deposited material appeared on the gold surface. Our interpretation of these images assumes that they are formed by chemically adsorbed probe DNA molecules, which under these conditions most likely assume a random/coiled conformation. Similar topographic features were reported by Holmberg et al. for single-stranded DNA immobilized on a gold surface^31^. Sealing the probe DNA layer with MCH thiol (see Fig. 3B) did not significantly change the surface morphology; irregular islets are still visible. However, definite changes were observed after exposing the test (Au/probe DNA/MCH) for 2 h to the sample containing the synthetic target DNA fully complementary to the probe DNA (see Fig. 3C). It should be stressed that, for all the probe DNA sequences and fully complementary synthetic target DNA sequences tested, the AFM response was the same. In this case, stretched strands are visible on the surface, which may be the result of hybridization between the probe DNA and the target DNA, which are fully complementary. Hybridization may force randomly oriented probe DNA strands to unfold and facilitate base pairing, thereby causing the molecules to change their conformation from randomly coiled to partially or fully stretched. As a result, fragments of hybridized DNA are exposed on the surface of the film. This scenario seems to be reasonable if one analyzes the morphology of the surface film after exposure to samples that contained DNA fragments with *BRCA1* mutation capable of hybridizing with the probe DNA dedicated to them. As can be seen in Fig. 3D–G, in these cases as well fragments of stretched DNA strands are visible on the surface of the film. Nevertheless, the frequency of their occurrence is lower than with the target DNA, and the observed fragments are usually shorter, which may be due to the fact that the DNA fragments present in the clinical samples differed in length from the probe DNA, making the base match only partial. On the other hand, after exposure to the samples containing DNA fragments without the *BRCA1* mutation, which had a limited ability to hybridize with the probe DNA, stretched strands did not appear. The AFM images presented in Fig. 3H, I clearly show that in this case we are dealing with irregular aggregates. Obviously, the above analysis of the AFM images provides only a purely qualitative comparison. Nevertheless, it clearly shows a noticeable difference in the morphology of the surface films, depending on the degree of complementarity and the ability to hybridize individual samples to the probe DNA. Moreover, the AFM data seem to correlate with the results obtained from the SPR and QCM-D.

### Identification of BRCA1 gene mutations in clinical samples

To verify the functionality of the procedure developed for identifying *BRCA1* gene mutations, studies were performed using clinical samples containing DNA. In this part of the studies, 22 different clinical samples collected from patients were used: 17 samples from patients with various *BRCA1* gene mutation variants (5370C > T: × 1 sample; 5382insC: × 10 samples; c.4035delA: × 2 samples; 185delAG: × 1 sample and 3819del5GTAAA: × 3 samples) and 5 samples from patients without any *BRCA1* mutation. The experiments were performed using *BRCA1* mutated gene sequences as the probe DNA. As can be seen from Table 4, the genotests with SPR and QCM detection performed perfectly as predictive tests to confirm or exclude the presence of specific mutations in an analyzed sample. The compatibility of the results obtained by these procedures and the gold standard-PCR is very good; ambiguous results were obtained in just two cases. In addition, the testing method presented detects missense single-nucleotide (SNP) mutations (class I (C/T conversions)), which are challenging for various screening technologies, including HRM. In contrast, several-nucleotide deletions and insertions are very easily detected by screening methods such as HRM, as they clearly destabilize the DNA structure^32^. There are four classes of SNPs in the human genome^33–35^. In the current study, we were able to distinguish samples in which there were deletions and insertions on the basis of the two measurement methods (QCM-D, SPR). And in the case of SNP 5370C > T it was possible to distinguish between the two genotypes. Importantly, polymorphisms of this type account for 64% of all SNPs in the human genome^33–35^. The specificity of the developed *BRCA1* mutation tests was determined on the basis of the negative samples analysis and was equalled 80% and 100% (4/5 and 5/5) for QCM-D and SPR tests, respectively. In turn, the selectivity calculated from the analysis the positive samples as a ratio of correct result/total no. of positive samples was equalled 94.1% and 100% (16/17 and 17/17) for QCM-D and SPR tests, respectively. The conventional methods applied in BRCA1 gene mutation identification are characterized by relatively broad range of the selectivity: 100% for enzymatic mutation detection (EMD), 50–96% for single-strand conformation polymorphism (SSCP), 88–91% for two-dimensional gene scanning (TDGS), 76% for conformation-sensitive gel electrophoresis (CSGE), 75% for protein truncation test (PTT), and 58% for micronucleus test (MNT)^26^. Whilst, the specificities of these methods are close to 100%, except for MNT. Taking into account the above information, it can be concluded that the developed SPR and QCM-D tests for *BRCA1* mutation identification can be a very good alternative and can be successfully used in diagnostics.

**Table 4:** Results of genotyping by PCR; SPR and QCM-D performed using *BRCA1* mutated gene sequences as the probe DNA.

Mutation	Sample	Detection		
		QCM-D	SPR	PCR
5370C > T	Mutation			
	5417/21	Ambiguous	Positive	Positive
	Without mutation			
	5297/21	Negative	Negative	Negative
	5623/21	Negative	Negative	Negative
	5528/21	Negative	Negative	Negative
	5577/21	Negative	Negative	Negative
	5615/21	Negative	Negative	Negative
5382insC	Mutation			
	5675/21	Positive	Positive	Positive
	5398/21	Positive	Positive	Positive
	2225/21	Positive	Positive	Positive
	5714/21	Positive	Positive	Positive
	5675/21	Positive	positive	Positive
	5252/21	Positive	Positive	Positive
	5187/21	Positive	Positive	Positive
	4937/21	Positive	Positive	Positive
	4930/21	Positive	Positive	Positive
	4928/21	Positive	Positive	Positive
	Without mutation			
	5297/21	Negative	Negative	Negative
	5623/21	Ambiguous	Negative	Negative
	5528/21	Negative	Negative	Negative
	5577/21	Negative	Negative	Negative
	5615/21	Negative	Negative	Negative
c.4035delA	Mutation			
	1848/21	Positive	Positive	Positive
	2942/21	Positive	Positive	Positive
	Without mutation			
	5297/21	Negative	Negative	Negative
	5623/21	Negative	Negative	Negative
	5528/21	Negative	Negative	Negative
	5577/21	Negative	Negative	Negative
	5615/21	Negative	Negative	Negative
185delAG	Mutation			
	2342	Positive	Positive	Positive
	Without mutation			
	5297/21	Negative	Negative	Negative
	5623/21	Negative	Negative	Negative
	5528/21	Negative	negative	Negative
	5577/21	Negative	Negative	Negative
	5615/21	Negative	Negative	Negative
3819del5GTAAA	Mutation			
	1606/21	Positive	Positive	Positive
	899/21	Positive	Positive	Positive
	901/21	Positive	Positive	Positive
	Without mutation			
	5297/21	Negative	Negative	Negative
	5623/21	Negative	Negative	Negative

## Conclusions

The paper demonstrates a novel, fast and cheap strategy for identifying *BRCA1* mutations. The detection was based on the morphological changes that took place in the sensing layer during a hybridization process with DNA fragments with and without a *BRCA1* mutation. Those changes were directly associated with the efficiency of the hybridization process and were also confirmed by visualizing the sensing layers with AFM, where the morphology of the surface films varied depending on the degree of complementarity and the ability to hybridize individual samples to the probe DNA. To our best knowledge, this was the first time morphological changes in the double-stranded DNA layer formed during the hybridization process were detected using the SPR and QCM-D techniques. The research carried out with the input of the clinical samples (17 with *BRCA1* mutation and 5 without), proved that the proposed protocol has great potential for identifying *BRCA1* mutations of the deletion type (one or several nucleotide bases), the insertion type, and the most difficult type—missense single-nucleotide (SNPs). Moreover, detection by means of SPR and QCM-D detectors is largely automated, which bodes well for developing point-of-care diagnostic devices. We believe this simple approach has great potential in medical diagnostics and will be easily adapted to detect other gene mutations using the oligonucleotide probe sequence. This will open new doors for fast-track early diagnostics, treatment monitoring, and screening activities in the general population.

## Supplementary Information

Supplementary Information.

## Data Availability

All data generated or analysed during this study are included in this published article (and its Supplementary Information files).
