# Supplementary material for: New, fast and cheap prediction tests for BRCA1 gene mutations identification in clinical samples
Source: Sci Rep. 2023 May 5;13:7316. doi: 10.1038/s41598-023-34588-9 (PMC10163215; doi:10.1038/s41598-023-34588-9)
Supplement: Supplementary file 1 — Supplementary Information. [file 41598_2023_34588_MOESM1_ESM.docx]

**Supporting Information**

**for**

**New, fast and cheap prediction tests for *BRCA1* gene mutations identification in clinical samples**

Aleksandra Gajda-Walczak,^a^ Agnieszka Potęga,^b^ Agata Kowalczyk,^a^ Slawomir Sek,^c^ Sebastian Zięba,^d^ Artur Kowalik,^d,e^ Andrzej Kudelski,^a^ Anna M. Nowicka^a*^

^a^Faculty of Chemistry, University of Warsaw, Pasteura 1 Str., 02-093 Warsaw, Poland

^b^Department of Pharmaceutical Technology and Biochemistry, Faculty of Chemistry, Gdańsk University of Technology, Narutowicza 11/12 Str., 80-233 Gdańsk, Poland

^c^Faculty of Chemistry, Biological and Chemical Research Centre, University of Warsaw, Żwirki i Wigury 101 Str., 02-089 Warsaw, Poland

^d^Molecular Diagnostics, Holy Cross Cancer Center, Stefana Artwińskiego 3 Str., 25-734 Kielce, Poland

^e^Division of Medical Biology, Institute of Biology, Jan Kochanowski University, Uniwersytecka 7 Str., 25-406 Kielce, Poland

*Corresponding author: Anna M. Nowicka, [anowicka@chem.uw.edu.pl](mailto:anowicka@chem.uw.edu.pl)


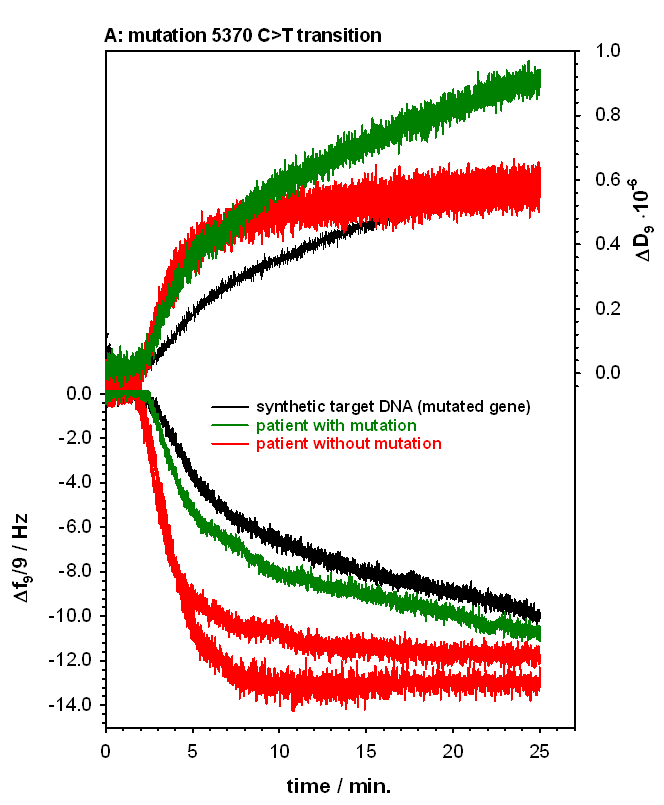


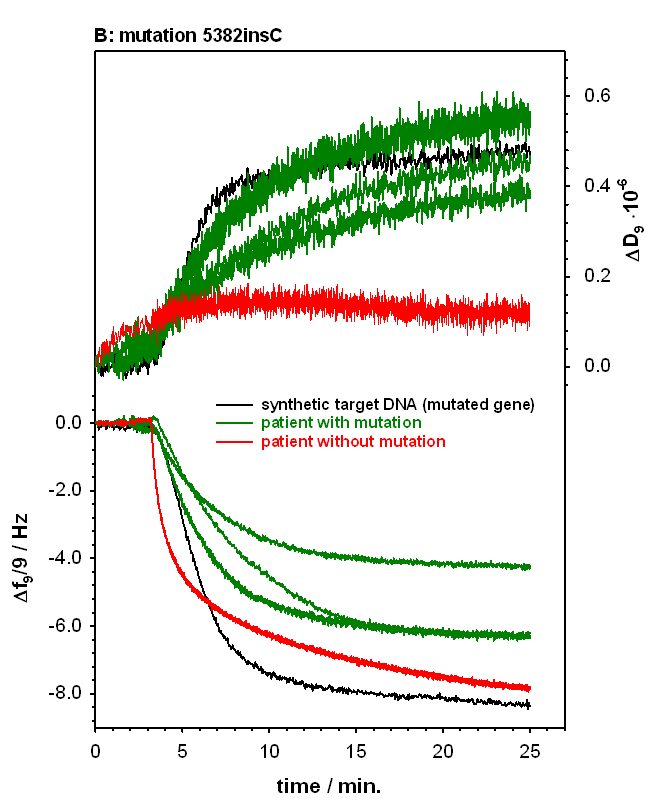


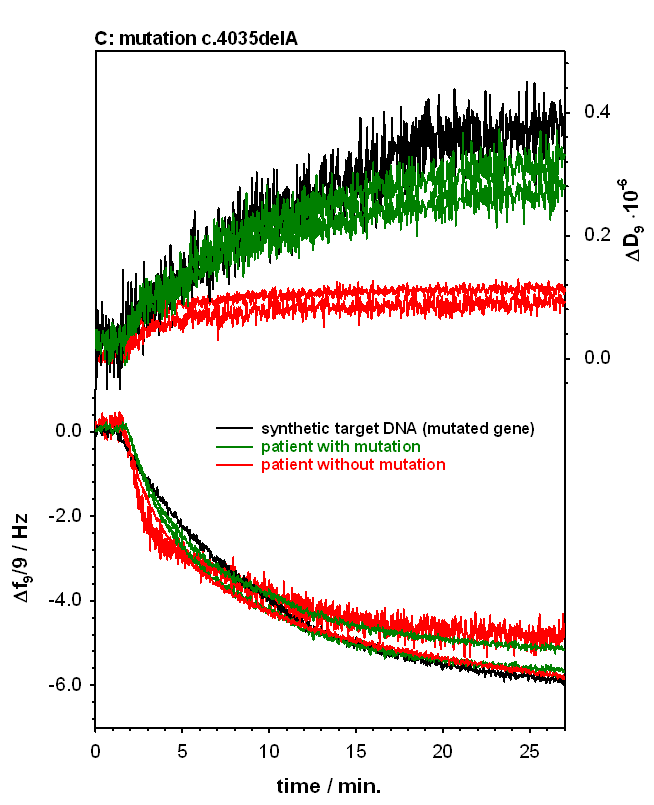


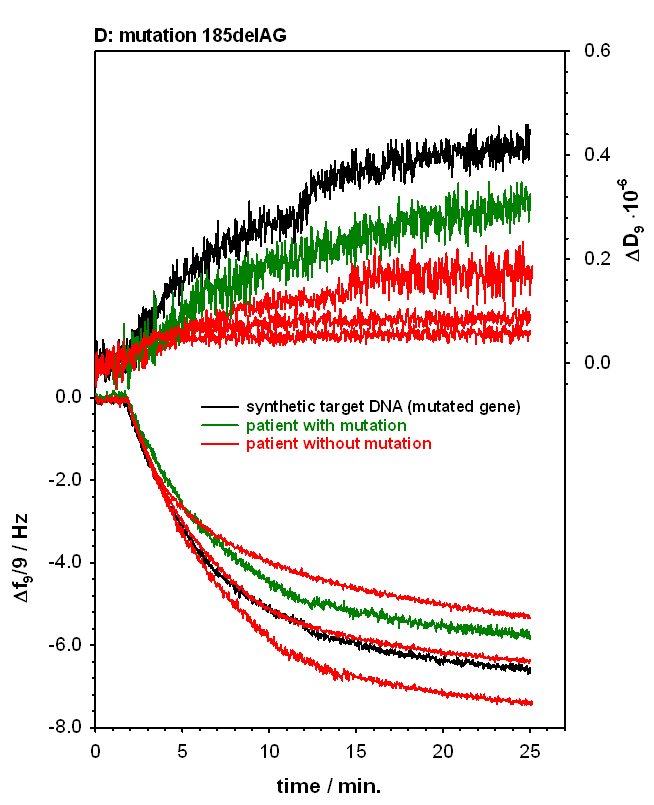


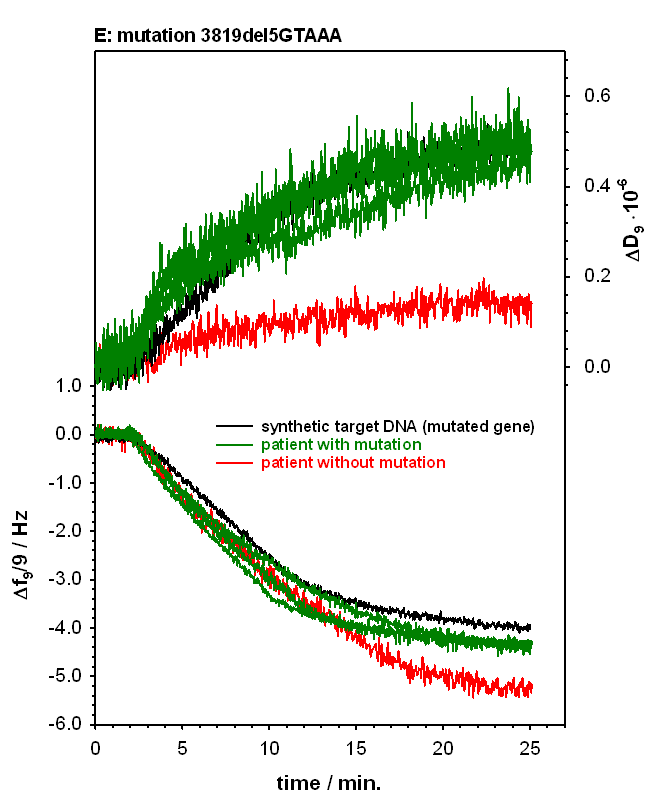


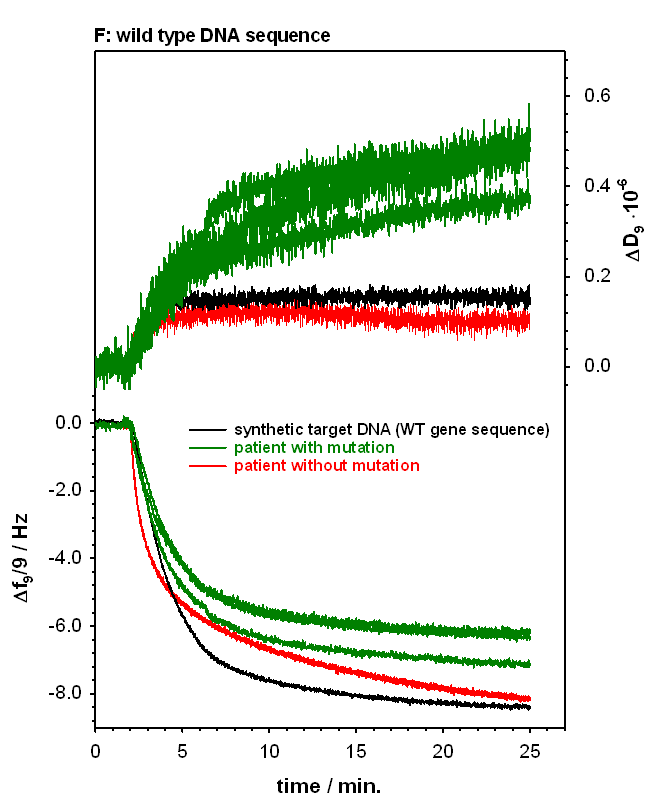


**Figure 1S.** The frequency (Δ*f*) and dissipation (Δ*D*) changes obtained during the hybridization process with target DNA sequences: synthetic mutated *BRCA1* gene sequence (black lines), sequences obtained from patients with (green lines) and without (red lines) appropriate mutations (A-E) and wild type DNA sequence (F). Experimental conditions: 0.01 M PBS with the addition of 1 mM EDTA and 1 M NaCl; *C*_probe DNA_ = 100 nM (*V*_droplet_ = 100 μL, *t* = 2 h); *C*_MCH_ = 1.0 μM (*V*_droplet_ = 100 μL, *t* = 1 h); *C*_synthetic target DNA_ = 100 nM (0.15 ng⋅μL^−1^) or 200-fold diluted clinical sample (*C*_DNA in clinical samples_ = 0.13 ng⋅μL^−1^).
